# Integration of Inflammation-Immune Factors to Build Prognostic Model Predictive of Prognosis and Minimal Residual Disease for Hepatocellular Carcinoma

**DOI:** 10.3389/fonc.2022.893268

**Published:** 2022-06-08

**Authors:** Xin Xu, Ao Huang, De-Zhen Guo, Yu-Peng Wang, Shi-Yu Zhang, Jia-Yan Yan, Xin-Yu Wang, Ya Cao, Jia Fan, Jian Zhou, Xiu-Tao Fu, Ying-Hong Shi

**Affiliations:** ^1^ Liver Cancer Institute, Zhongshan Hospital, Fudan University and Key Laboratory of Carcinogenesis and Cancer Invasion (Fudan University), Ministry of Education, Shanghai, China; ^2^ Department of Liver Oncology, Zhongshan Hospital, Fudan University, Shanghai, China; ^3^ Department of Liver Surgery and Transplantation, Zhongshan Hospital, Fudan University, Shanghai, China; ^4^ Cancer Research Institute, Xiangya School of Medicine, Central South University; Key Laboratory of Carcinogenesis and Cancer Invasion, Ministry of Education, Xiangya Hospital, Central South University, Changsha, China; ^5^ Institute of Biomedical Sciences, Fudan University, Shanghai, China; ^6^ State Key Laboratory of Genetic Engineering, Fudan University, Shanghai, China

**Keywords:** prognostic model, inflammation, immunity, hepatocellular carcinoma, prognosis

## Abstract

**Background:**

Tumor recurrence after hepatectomy is high for hepatocellular carcinoma (HCC), and minimal residual disease (MRD) could be the underlying mechanism. A predictive model for recurrence and presence of MRD is needed.

**Methods:**

Common inflammation-immune factors were reviewed and selected to construct novel models. The model consisting of preoperative aspartate aminotransferase, C-reactive protein, and lymphocyte count, named ACLR, was selected and evaluated for clinical significance.

**Results:**

Among the nine novel inflammation-immune models, ACLR showed the highest accuracy for overall survival (OS) and time to recurrence (TTR). At the optimal cutoff value of 80, patients with high ACLR (> 80) had larger tumor size, higher Edmondson’s grade, more vascular invasion, advanced tumor stage, and poorer survival than those with low ACLR (≤ 80) in the training cohort (5-year OS: 43.3% vs. 80.1%, *P* < 0.0001; 5-year TTR: 74.9% vs. 45.3%, *P* < 0.0001). Multivariate Cox analysis identified ACLR as an independent risk factor for OS [hazard ratio (HR) = 2.22, *P* < 0.001] and TTR (HR = 2.36, *P* < 0.001). Such clinical significance and prognostic value were verified in validation cohort. ACLR outperformed extant models, showing the highest area under receiver operating characteristics curve for 1-, 3-, and 5-year OS (0.737, 0.719, and 0.708) and 1-, 3-, and 5-year TTR (0.696, 0.650, and 0.629). High ACLR correlated with early recurrence (*P* < 0.001) and extremely early recurrence (*P* < 0.001). In patients with high ACLR, wide resection margin might confer survival benefit by decreasing recurrence (median TTR, 25.5 vs. 11.4 months; *P* = 0.037).

**Conclusions:**

The novel inflammation-immune model, ACLR, could effectively predict prognosis, and the presence of MRD before hepatectomy and might guide the decision on resection margin for patients with HCC.

## Introduction

Hepatocellular carcinoma (HCC) is the sixth most common cancer and the fourth leading cause of cancer-related death worldwide. In China, where the hepatitis B infection rate is high, HCC ranks fourth in cancer morbidity and second in cancer mortality (1, 2). Despite advances in diagnosis and treatment, the prognosis of HCC remains dismal, and tumor recurrence stands as the major cause of poor outcomes (3, 4). Therefore, it is of unmet need to develop robust biomarkers and prognostic models to distinguish patients with HCC with high recurrence risk and poor survival.

It is well established that systemic inflammation and immunity play vital roles in the carcinogenesis and metastasis of cancers (5). In fact, emerging evidence has demonstrated the prognostic value of systemic inflammation and immune markers, including neutrophil (6, 7), lymphocyte (8), platelet (9), and C-reactive protein (CRP) (10, 11), in various cancers including HCC. Moreover, chronic liver inflammation, which commonly results in hepatic function damage, also participates in tumorigenesis (12, 13), and factors such as aspartate aminotransferase (AST) (14) and albumin (15) are found to be predictive of prognosis. On the basis of these markers, several models have been developed to predict outcomes, including Glasgow prognostic score (GPS) (16), modified GPS (mGPS) (17), prognostic nutritional index (PNI) (18), neutrophil-to-lymphocyte ratio (NLR) (19), platelet-to-lymphocyte ratio (PLR) (20), and systemic immune-inflammation index (SII) (21). Moreover, models that combine systemic inflammation and chronic liver inflammation indexes, such as aspartate aminotransferase–to-platelet ratio index (APRI) (22) and aspartate aminotransferase–to-lymphocyte ratio index (ALRI) (23), have also been reported.

However, the prognostic value of the current existing models is not fully satisfactory because most models were constructed with only two inflammation or immune factors, and it is proposed that the combination of three markers might achieve better prediction efficiency. Moreover, few models have addressed the underlying issue of tumor recurrence, especially considering that minimal residual disease (MRD) has been increasingly regarded as the fundamental reason for tumor recurrence. In this study, we thoroughly reviewed currently available models and scrutinized the factors that they had used. We then constructed novel models using different combinations of those factors, evaluated their predicting values, and chose the one with the highest predictive accuracy. The prognostic value and clinical implication of the selected model were further assessed and validated.

## Methods

### Patient Inclusion

Patients with HCC who underwent curative hepatectomy at the Zhongshan Hospital Fudan University from January 2013 to December 2015 were retrospectively analyzed. The inclusion criteria were as follows: (a) HCC confirmed by pathology, (b) no preoperative anti-tumor treatment, and (c) R0 resection. The exclusion criteria were as follows: (a) incomplete clinicopathological information or follow-up data, (b) presence or history of malignancies in extrahepatic organs, and (c) had preoperative anti-tumor treatment. Clinicopathological data and preoperative laboratory test results of all included patients were retrieved from medical documents. The tumor stage was assessed according to the Barcelona Clinic Liver Cancer (BCLC) stage (24), American Joint Committee on Cancer (AJCC) stage (25), and China Liver Cancer Staging (CNLC) (26). Edmondson’s grading system was used to grade tumor differentiation (27). The Child-Pugh score system was applied to evaluate liver function. The present study was conducted in accordance with the Strengthening the Reporting of Observational Studies in Epidemiology (STROBE) recommendations (28). This study was approved by the Zhongshan Hospital Research Ethics Committee (No. B2020-427).

### Inflammation-Immune Models

Factors that constituted previously reported inflammation-immune prognostic models were analyzed, and frequently used key factors were selected for further model construction. The factors were first formulated into different combinations, and the one with the highest accuracy to predict outcomes in the training cohort was chosen and tested in the validation cohort. The one that had consistent and the highest prognostic value in both cohorts was finally identified as our goal model.

### Follow-Up

Postoperative follow-up was first performed 1 month after hepatectomy and then every 3 months until death or loss to follow-up. At each follow-up, laboratory tests including serum α-fetoprotein (AFP), chest X-ray, and abdominal ultrasonography were given to each patient. If recurrence was suspected, then an abdominal computed tomography (CT) or magnetic resonance imaging (MRI) scan was performed for differential diagnosis. Moreover, chest CT was applied to diagnose pulmonary metastasis, and positron emission tomography–CT (PET-CT) was used when extrahepatic metastases were suspected. Time to recurrence (TTR) was defined as the time from hepatectomy to recurrence. Overall survival (OS) was defined as the interval between hepatectomy and death or the end of the study. The last follow-up was made on October 1, 2020.

### Statistical Analysis

Statistical analyses were performed using R 4.0.4 software (R Foundation for Statistical Computing, Vienna, Austria). Continuous variables were summarized as median (interquartile range) or mean (standard deviation), and Wilcoxon rank sum test or t-test was applied to compare the differences between the two groups, respectively. Categorical variables were shown as frequency (percentage) and compared *via* chi-square test or Fisher’s exact test. The optimal cutoff values of inflammation-immune models were defined *via* the “surminer” package using R. Area under the curve (AUC) of receiver operating characteristics (ROC) and C-index were used to assess the predictive values for OS, TTR, and 90-day recurrence. The cumulative survival and recurrence rates were calculated using the Kaplan–Meier method and the differences between the two groups were evaluated by the log-rank test. Univariate and multivariate analyses were performed using the Cox regression model and the hazard ratio (HR) and 95% confidence interval (CI) were calculated. *P*-value less than 0.05 was considered statistically significant.

## Results

### Characteristics of Patients

A total of 1,031 patients with HCC were retrieved, and, among them, 617 patients treated between 2013 and 2014 constituted the training cohort, whereas 414 patients treated in 2015 were enrolled in the validation cohort. The baseline characteristics of patients in the two cohorts were comparable, and no significant differences were identified, except for gender and AFP (**Supplementary Table 1**). The median follow-up time was 60.9 months (0.5–90.6 months) in the training cohort and 54.8 months (0.4–69.9 months) in the validation cohort. During the follow-up, 305 (29.6%) patients died and 581 (56.4%) patients experienced tumor recurrence. There were no significant differences between training cohort and validation cohort regarding 5-year survival rate (29.8% vs. 27.5%, *P* = 0.430) and 5-year recurrence rate (54.6% vs. 58.9%, *P* = 0.170).

### Construction and Selection of Novel Inflammation-Immune Model-ACLR

Thorough scrutiny of the existing inflammation-immune models (GPS, mGPS, PNI, NLR, PLR, SII, APRI, and ALRI; **Supplementary Table 2**) identified the five most frequently used factors: lymphocyte and albumin positively correlated with prognosis, whereas neutrophil, CRP, and AST negatively correlated with prognosis. These markers were subsequently selected, and nine novel inflammation-immune models were constructed (**Supplementary Figure 1**). The combination of these factors for each model was shown in **Supplementary Table 3**. For example, ACLR was calculated as follows: AST (U/L) × CRP (mg/L)/lymphocyte count (×10^9^/L).

For each model, the optimal cutoff value was obtained as described above. The C-index and AUC of these models for predicting 1-, 3-, and 5-year OS and TTR were shown in **Table 1**. Among the nine models, ACLR harbored the highest predictive value for both survival and recurrence (cutoff value: 80; C-index for OS: 0.660; TTR: 0.608) in the training cohort. Then, the predictive performance was further assessed in the validation cohort with the same cutoff. Consistently, ACLR bears the highest predictive significance with a C-index of 0.729 and 0.642 for OS and TTR, respectively. According to these findings, ACLR was chosen as the target model in this study, and its predictive value and prognostic potential were further assessed in patients with HCC.

**Table 1:** C-indexes and AUCs of the nine models in the training and validation cohort.

Model	Cutoff	OS in training (n = 617)				TTR in training (n = 617)				OS in validation (n = 414)				TTR in validation (n = 414)			
		C-index	1-year AUC	3-year AUC	5-year AUC	C-index	1-year AUC	3-year AUC	5-year AUC	C-index	1-year AUC	3-year AUC	5-year AUC	C-index	1-year AUC	3-year AUC	5-year AUC
ACLR	80.0	0.66	70.0	68.8	68.5	0.608	67.3	63.6	62.3	0.729	78.2	76.7	74.5	0.642	73.2	67.5	64.1
NALR	151.5	0.60	68.2	62.5	60.7	0.55	58.4	56.0	55.7	0.619	64.3	65.1	61.9	0.565	60.9	57.6	54.5
NCLR	6.24	0.652	69.1	67.8	67.8	0.594	65.4	61.9	60.4	0.714	76.6	76.4	72.8	0.625	72.8	64.7	62.8
ACBR	3.72	0.655	71.0	69.8	67.5	0.599	65.2	62.4	61.2	0.722	77.2	76.5	72.6	0.635	70.8	66.8	63.3
NABR	5.93	0.588	64.8	61.2	59.3	0.551	58.4	56.0	55.4	0.608	60.3	63.6	61.8	0.570	58.9	59.7	57.3
NCBR	0.195	0.644	68.3	67.9	67.4	0.595	65.9	61.9	60.8	0.693	74.2	73.3	71.0	0.622	72.4	64.1	63.6
ABLR	1.95	0.548	59.3	57.0	55.6	0.525	52.5	53.4	53.6	0.538	53.8	54.3	52.8	0.528	50.9	54.9	52.9
NBLR	0.030	0.521	55.1	53.1	51.7	0.509	53.1	51.1	50.0	0.512	50.2	51.8	51.4	0.500	51.0	49.8	48.0
CBLR	0.129	0.630	67.0	66.3	65.4	0.578	61.4	61.0	59.3	0.657	64.7	69.2	63.5	0.583	65.1	59.0	55.6

### High ACLR Correlated With Advanced Clinicopathological Features

**Figure 1:**
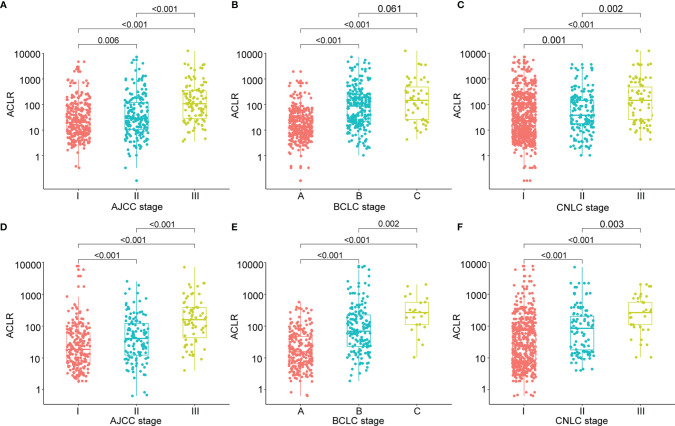
Clinical significance of ACLR in patients with HCC from the training cohort and validation cohort. **(A–C)** Scattergrams of ACLR according to **(A)** AJCC stage, **(B)** BCLC stage, and **(C)** CNLC stage in patients with HCC from the training cohort. **(D–F)** Scattergrams of ACLR according to **(D)** AJCC stage, **(E)** BCLC stage, and **(F)** CNLC stage in patients with HCC from the validation cohort.

We then first investigated the correlation between ACLR and clinicopathological factors of patients with HCC. In the training cohort, patients with high ACLR (> 80) tended to have tumors with advanced clinical phenotypes including larger tumor size (*P* < 0.001), higher Edmondson’s grade (*P* < 0.001), more vascular invasion (*P* < 0.001), and higher AFP level (*P* = 0.004) (**Supplementary Figures 2A–D**). Correspondingly, patients with high ACLR were also diagnosed at advanced AJCC stage (*P* < 0.001), BCLC stage (*P* < 0.001), and CNLC stage (*P* < 0.001; **Figures 1A–C**).

To validate the clinical significance of ACLR, we performed correlation analysis of ACLR with clinicopathological factors in patients with HCC from the validation cohort. Similarly, high ACLR was also associated with larger tumor size (*P* < 0.001), higher Edmondson’s grade (*P* < 0.001), vascular invasion (*P* < 0.001), higher AFP level (*P* = 0.038) (**Supplementary Figures 3A–D**), advanced AJCC stage (*P* < 0.001), BCLC stage (*P* < 0.001), and CNLC stage (*P* < 0.001) (**Figures 1D–F**).

### High ACLR Predicted an Unfavorable Prognosis of HCC

**Figure 2:**
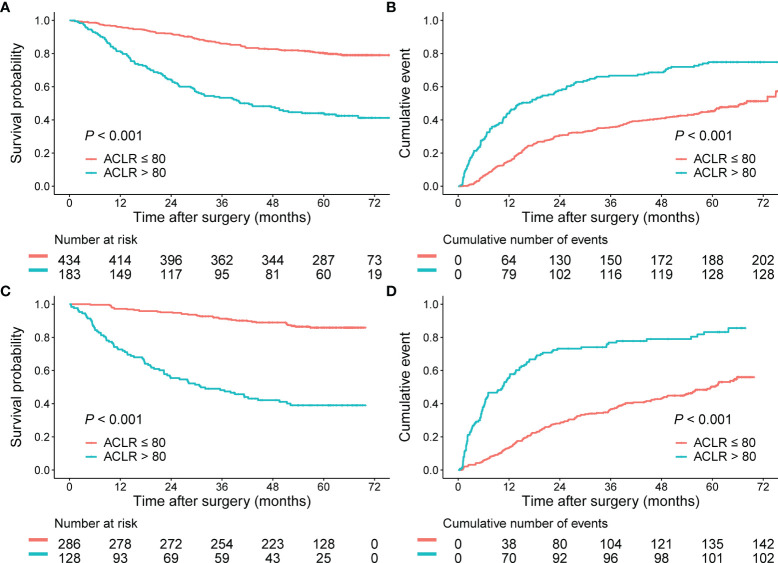
Prognostic performance of ACLR in patients with HCC from the training cohort and validation cohort. **(A, B)** Kaplan–Meier analysis of OS **(A)** and recurrence rates **(B)** for patients with HCC stratified by ACLR (cutoff = 80) from the training cohort. **(C, D)** Kaplan–Meier analysis of OS **(C)** and recurrence rates **(D)** for patients with HCC stratified by ACLR (cutoff = 80) from the validation cohort.

To assess the value of preoperative ACLR as a prognostic biomarker, Kaplan–Meier analysis was performed. Patients in the training cohort were divided into two groups based on the optimal cutoff value of 80 for ACLR. Consistent with the result that high ACLR correlated with advanced HCCs, patients with high ACLR demonstrated significantly poorer survival than patients with low ACLR (≤ 80) (n: 183 vs. 434; 1-, 3-, and 5-year OS: 80.9%, 52.8%, and 43.3% vs. 95.8%, 85.8%, and 80.1%; median OS: 40.3 months vs. unreached; *P* < 0.001; **Figure 2A**). Correspondingly, the 1-, 3-, and 5-year cumulative recurrence rates in patients with high ACLR were 45.1%, 67.4%, and 74.9%, respectively, with a median TTR of 15.0 months, which were significantly poorer than those of patients with low ACLR (1-, 3-, and 5-year recurrence rates: 15.2%, 35.8%, and 45.3%; median TTR: 67.5 months; *P* < 0.001; **Figure 2B**).

Univariate Cox regression analysis identified that AFP level, tumor size, tumor number, Edmondson’s grade, vascular invasion, GPS, NLR, PLR, SII, and ACLR were associated with both OS and TTR (**Table 2**). Subsequently, these factors were taken into multivariate Cox analysis and only tumor size, tumor number, vascular invasion, and ACLR were found to be independent risk factors for both OS and TTR (ACLR for OS: HR = 2.22, 95% CI, 1.50–3.27, *P* < 0.001; ACLR for TTR: HR = 2.36, 95% CI, 1.89–2.94, *P* < 0.001; **Table 2**).

**Table 2:** Univariate and multivariate analyses in the training cohort.

Variables	OS				TTR			
	Univariate analysis		Multivariate analysis		Univariate analysis		Multivariate analysis	
	HR (95% CI)	*P*-value	HR (95% CI)	*P*-value	HR (95% CI)	*P*-value	HR (95% CI)	*P*-value
Age, y (50: >50)	0.82 (0.61–1.10)	0.180			0.82 (0.66–1.03)	0.084	0.79 (0.63–0.98)	0.034
Gender (male: female)	1.15 (0.81–1.63)	0.448			0.98 (0.74–1.29)	0.887		
HBsAg (negative: positive)	0.95 (0.66–1.38)	0.805			1.19 (0.88–1.60)	0.251		
Cirrhosis (no: yes)	1.15 (0.86–1.54)	0.340			1.15 (0.92–1.43)	0.215		
Child-Pugh stage (A: B)	0 (0-Inf)	0.994			0 (0-Inf)	0.993		
AFP, ng/ml (≤20: >20)	1.68 (1.23–2.28)	0.001	1.24 (0.89–1.73)	0.199	1.57 (1.25–1.97)	<0.001	1.30 (1.02–1.66)	0.032
Tumor size, cm (≤5: >5)	3.57 (2.67–4.77)	<0.001	1.67 (1.16–2.41)	0.006	2.09 (1.69–2.60)	<0.001	1.31 (1.00–1.70)	0.048
Tumor number (solitary: multiple)	2.29 (1.72–3.05)	<0.001	1.96 (1.46–2.62)	<0.001	1.93 (1.54–2.42)	<0.001	1.74 (1.38–2.21)	<0.001
Edmondson grade (I/II: III/IV)	1.90 (1.43–2.53)	<0.001	1.26 (0.92–1.73)	0.157	1.36 (1.10–1.69)	0.005	1.05 (0.82–1.33)	0.700
Vascular invasion (no: yes)	3.01 (2.26–4.01)	<0.001	1.85 (1.36–2.53)	<0.001	1.81 (1.46–2.26)	<0.001	1.37 (1.08–1.74)	0.01
GPS	2.13 (1.68–2.70)	<0.001	1.12 (0.83–1.52)	0.462	1.51 (1.23–1.86)	<0.001	0.93 (0.72–1.21)	0.593
NLR (≤2.7: >2.7)	2.32 (1.72–3.13)	<0.001	1.34 (0.90–2.00)	0.155	1.52 (1.19–1.94)	0.001	1.12 (0.80–1.55)	0.51
PLR (≤133.1: >133.1)	2.42 (1.77–3.31)	<0.001	0.95 (0.6–1.49)	0.814	1.84 (1.42–2.38)	<0.001	1.24 (0.84–1.81)	0.276
SII (≤523.8: >523.8)	2.70 (1.98–3.67)	<0.001	1.11 (0.65–1.89)	0.705	1.67 (1.28–2.17)	<0.001	0.88 (0.56–1.39)	0.589
ACLR (≤80: >80)	3.92 (2.95–5.22)	<0.001	2.22 (1.50–3.27)	<0.001	2.36 (1.89–2.94)	<0.001	1.94 (1.44–2.61)	<0.001

### Consistent Prognostic Value of ACLR in the Validation Cohort

Kaplan–Meier survival analysis and Cox regression analysis were then performed in the validation cohort. Patients with high ACLR showed significantly shorter OS than patients with ACLR ≤ 80 (n: 128 vs. 286; 1-, 3-, and 5-year OS: 71.9%, 47.4%, and 39.0% vs. 96.9%, 91.2%, and 85.9%; median OS: 31.0 months vs. unreached; *P* < 0.001; **Figure 2C**). Moreover, the cumulative recurrence rates for patients with high ACLR dramatically increased when compared with those for patients with low ACLR (1-, 3-, and 5-year recurrence rates: 56.2%, 77.8%, and 83.2% vs. 13.7%, 37.0%, and 50.3%; median TTR: 10.5 vs. 59.7 months; *P* < 0.001; **Figure 2D**). In the Cox regression model, both univariate and multivariate analysis verified that ACLR was an independent risk factor for both OS (HR = 4.16; 95% CI, 2.59–6.69; *P* < 0.001) and TTR (HR = 2.19; 95% CI, 1.59–3.03, *P* < 0.001; **Supplementary Table 4**) in patients with HCC of the validation cohort.

### ACLR Outperformed Extant Inflammation-Immune Models on Prognostication

**Figure 3:**
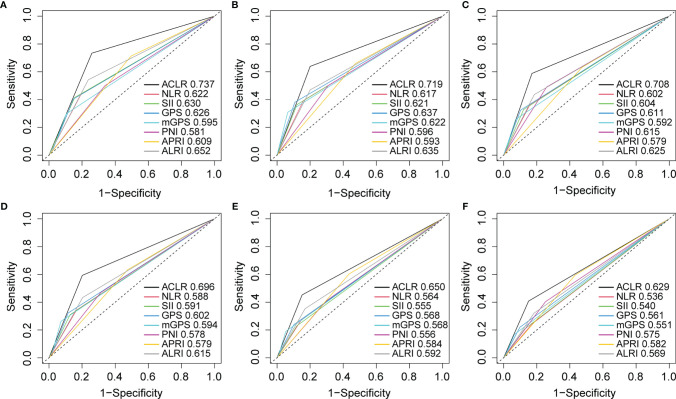
Receiver operating characteristic (ROC) curves analysis to compare the predictive value of ACLR with other inflammation-immune models. **(A–C)** ROC curves of ACLR and other inflammation-immune models for **(A)** 1-, **(B)** 3-, and **(C)** 5-year OS in the whole cohort. **(D–F)** ROC curves of ACLR and other inflammation-immune models for **(D)** 1-, **(E)** 3-, and **(F)** 5-year TTR in the whole cohort.

To further explore the prognostic value of ACLR, we compared ACLR with recently reported inflammation-immune models, including GPS, mGPS, PNI, NLR, PLR, SII, APRI, and ALRI, in the whole cohort. ROC analysis demonstrated that ACLR possessed the highest predictive accuracy for survival with the AUCs for 1-, 3-, and 5-year OS of 0.737, 0.719, and 0.708, respectively (**Figures 3A–C**). Meanwhile, the AUCs for 1-, 3-, and 5-year TTR were 0.696, 0.650, and 0.629, respectively, which were also the highest among all selected models (**Figures 3D–F**).

### The Implication of ACLR for MRD and Resection Margin

**Figure 4:**
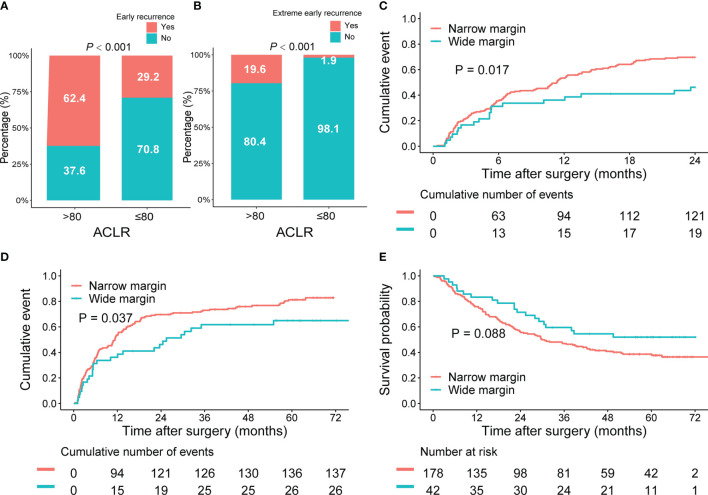
Implications of ACLR for early recurrence and resection margin for patients with HCC of the whole cohort. **(A, B)** Comparison of **(A)** early recurrence and **(B)** extreme early recurrence rates between patients with HCC with high and low ACLR. **(C–E)** Kaplan–Meier analysis of 2-year cumulative recurrence rate **(C)**, TTR **(D)**, and OS **(E)** for patients with HCC with high ACLR, stratified by the resection margin.

Early recurrence, commonly resulting from MRD and occurring within 2 years after hepatectomy, indicated dismal outcomes for patients with HCC. Effective identification of these patients and timely application of adjuvant therapy might eliminate the potential minimal residual tumor cells and improve survival (29). Thus, we explored whether ACLR could accurately identify patients with a high risk of early recurrence. Indeed, patients with high ACLR were associated with higher early recurrence rate (62.4% vs. 29.2%, *P* < 0.001, **Figure 4A**). Moreover, high ACLR was also found to be related to extremely early recurrence (3-month after hepatectomy; 19.6% vs. 1.9%, *P* < 0.001, **Figure 4B**). Considering the extremely early recurrence might derive from the rapid development of disseminated tumor cells around the resection margin—in other words, MRD (30–32), we further explored the association of ACLR with recurrence around the resection margin. Factually, patients with high ACLR had a much higher recurrence rate around the resection margin than those with low ACLR (6.8% vs. 1.1%, P < 0.001, **Supplementary Figure 5A**).

Subsequently, we investigated the implication of ACLR for resection margin in patients with HCC. Patients were classified into wide resection margin group (margin > 1 cm; n = 168) or narrow resection margin group (margin ≤ 1 cm; n = 592) accordingly (26, 33, 34). In patients with high ACLR, both 2-year recurrence rate (48.8% vs. 69.7%; *P* = 0.017; **Figure 4C**) and TTR (median TTR, 25.5 vs. 11.4 months; *P* = 0.037, **Figure 4D**) were significantly better in the wide margin group than those in the narrow margin group. Meanwhile, wide margin also conferred a significant trend of longer OS compared with the narrow margin group (median OS, unreached vs. 29.9 months; *P* = 0.088; **Figure 4E**). By contrast, in patients with low ACLR, 2-year recurrence rate (*P* = 0.660; **Supplementary Figure 4A**), TTR (*P* = 0.760; **Supplementary Figure 4B**), and OS (*P* = 0.480; **Supplementary Figure 4C**) were all comparable between patients with different margins. Consistently, we found that in patients with resection margin ≤ 1 cm, high ACLR indicated a higher risk of margin recurrence than those with low ACLR (7.2% vs. 1.0%; *P* < 0.001; **Supplementary Figure 5B**). However, in patients with surgical margin >1 cm, there was no difference in the margin recurrence risk between high and low ACLR patients (2.4% vs. 0.8%; *P* = 0.411; **Supplementary Figure 5C**).

## Discussion

Tumor recurrence remains the main obstacle to further improvement of survival in HCC. Accurate prediction of patients at high risk of recurrence before surgery, employment of proper resection margin during operation, and timely implementation of prophylactic treatment after hepatectomy would ensure a favorable outcome. Currently, few prognostic models have fulfilled such demand with acceptable accuracy, and novel models are still needed. Regarding this, we developed a new inflammation-immune model that could not only distinguish patients with high recurrence risk but also provide information possibly guiding the resection margin and choosing candidates for postoperative adjuvant therapy.

Different from previous studies that used fixed factors to construct models, we chose a novel strategy; we first analyzed the existing models and gathered the most common inflammation-immune factors. Then, we built nine models using the five common inflammation-immune factors with different combinations. This method was more comprehensive and objective because we had no presupposition which factors would be positively or negatively correlated with survival or recurrence. Only after comparing the prognostic value of all models that we had identified the combination of AST, CRP, and lymphocyte counts, namely, ACLR, as the target one with the best predictive performance in both the training and validation cohort. High ACLR was associated with advanced tumor characteristics and poorer prognosis than low ACLR. Multivariate Cox analyses also verified that high ACLR was an independent risk factor for both OS and TTR.

We also compared the newly built ACLR model with widely accepted prognostic ones, including GPS, NLR, PLR, and SII (16, 19–21). Although these models also showed satisfactory predictive efficiencies for both survival and recurrence in our cohort of patients, our model displayed some advantages, namely, the ability to predict early recurrence and indicate invasion margin. Notably, we found that high ACLR was strongly associated with early recurrence and extremely early recurrence. Early recurrence after radical resection is commonly considered the outcome of MRD, which could be hardly detected using conventional imaging and serum tumor biomarkers (30, 35, 36). Currently, liquid biopsy had been proposed as a promising tool for MRD prediction in HCC. However, liquid biopsy is generally performed postoperatively and our model could be a viable option for MRD prediction before surgery.

Another interesting finding is that ACLR correlated with resection margin. It was reported that a wider resection margin could remove potential MRD around the tumor and decrease the early recurrence of patients with HCC after curative hepatectomy (34). However, for patients with HCC with large tumors or multiple lesions, especially on a cirrhotic background, a wide margin may leave insufficient liver remnant and lead to liver failure (37). Therefore, surgical outcome and liver function should be well balanced *via* choosing suitable resection margins for different patients with HCC. In the present study, a wide resection margin (>1 cm) could significantly decrease tumor recurrence after hepatectomy compared with a narrow margin for patients with HCC with high ACLR. Considering the fact that high ACLR correlated with vascular invasion and more advanced tumor phenotype, it is easy to understand such a connection: HCC with advanced phenotypes is at high risk of tumor dissemination into the microvascular around the resection margin and a wide resection margin would eradicate such MRD and decrease early recurrence. Thus, ACLR might serve as a biomarker for surgical oncologists to better prepare hepatectomy—making a wide resection margin in patients with high ACLR not only to minimize tumor recurrence risk but also to avoid the unnecessary sacrifice of hepatic reserve in patients with low ACLR and liver cirrhosis.

ACLR consists of three factors of which the associations with tumor progression and outcomes of HCC were well established. As a protein mainly synthesized in the acute phase of inflammation, CRP is stimulated by cytokines such as interleukin 6 and produced in the liver (38). CRP takes part in a wide range of inflammatory processes and connects the innate immune system with the adaptive immune system (39). Emerging evidence showed that CRP may also participate in tumor progression and metastasis in patients with HCC (40, 41). Meanwhile, several studies suggested elevated CRP predicted poor prognosis in both cirrhotic patients and patients with HCC (10, 42, 43). AST reflects damaged liver function, and increased AST might also indicate the activity of hepatitis B or C virus and progression of tumor (44). Immune response to malignancy depends on lymphocyte population, and decreased lymphocyte might lead to impaired defense against malignancy. Several pieces of evidence have showed that reduced lymphocytic infiltration in tumor was an independent risk factor in patients with HCC (45, 46). The combination of AST, CRP, and lymphocyte counts simultaneously reflects liver function damage, systemic inflammation, and immune response of patients with HCC. All three processes could affect the outcomes of patients with HCC after curative resection.

This study has some limitations. First, although ACLR could differentiate patients with HCC with poor prognosis and show good predictive value on early recurrence and extreme early recurrence, its accuracy for long-term prognosis is still unsatisfactory. This might be attributed to the recent advances in immunotherapy and targeted therapy that have prolonged the survival after recurrence. Second, the inflammation-immune model was only validated in another independent cohort from our center. Further external validation is still needed. In addition, all patients included in this study were from China and the predominant etiology was HBV infection. The clinical significance of ACLR in HCC with other etiology needs further investigation.

## Conclusion

In conclusion, this study constructed a novel inflammation-immune model containing AST, CRP, and lymphocyte count, which not only could effectively predict prognosis and MRD for patients with HCC after curative resection but also might guide the clinical decision of optimal resection margin.

## Data Availability Statement

The raw data supporting the conclusions of this article will be made available by the authors, without undue reservation.

## Ethics Statement

The studies involving human participants were reviewed and approved by the Zhongshan Hospital Research Ethics Committee. Written informed consent for participation was not required for this study in accordance with the national legislation and the institutional requirements.

## Author Contributions

Y-HS, XX, and X-TF designed the study and wrote the manuscript. D-ZG and AH performed the analysis of the data. Y-PW, S-YZ, J-YY, and X-YW collected patients’ information and created the database. YC, JF, and JZ interpreted the data and revised the manuscript. All authors edited the manuscript. All authors contributed to the article and approved the submitted version.

## Funding

This study was jointly supported by the National Key R&D Program of China (2019YFC1315800, 2019YFC1315802), National Natural Science Foundation of China (No.82150004, 82073217, 81830102), Fudan University (IDF152064/014), Zhongshan Hospital Fudan University (2021ZSYQ09), Shanghai Municipal Key Clinical Specialty, the Clinical Research Fundation of Shanghai Municipal Health Commission (No. 20204Y0226) and the Projects from the Shanghai Science and Technology Commission (No. 19XD1401100).

## Conflict of Interest

The authors declare that the research was conducted in the absence of any commercial or financial relationships that could be construed as a potential conflict of interest.

The handling editor QL declared a shared parent affiliation with the authors XX, AH, DG, YW, SZ, JY, XW, JF, JZ, XF, YS at the time of review.

## Publisher’s Note

All claims expressed in this article are solely those of the authors and do not necessarily represent those of their affiliated organizations, or those of the publisher, the editors and the reviewers. Any product that may be evaluated in this article, or claim that may be made by its manufacturer, is not guaranteed or endorsed by the publisher.

## Abbreviations

AFP, alpha-fetoprotein; AJCC, American Joint Committee on Cancer; ALRI, aspartate aminotransferase–to-lymphocyte ratio index; APRI, aspartate aminotransferase–to-platelet ratio index; AST, aspartate aminotransferase; AUC, area under the curve; BCLC, Barcelona Clinic Liver Cancer; CI, confidence interval; CNLC, China Liver Cancer Staging; CRP, C-reactive protein; CT, computed tomography; GPS, Glasgow prognostic score; HCC, hepatocellular carcinoma; HR, hazard ratio; mGPS, modified Glasgow prognostic score; MRD, minimal residual disease; MRI, magnetic resonance imaging; NLR, neutrophil-to-lymphocyte ratio; OS, overall survival; PET-CT, positron emission tomography–computed tomography; PLR, platelet-to-lymphocyte ratio; PNI, prognostic nutritional index; ROC, receiver operating characteristics; SII, systemic immune-inflammation index; TTR, time to recurrence.
