# Supplementary material for: Integration of Inflammation-Immune Factors to Build Prognostic Model Predictive of Prognosis and Minimal Residual Disease for Hepatocellular Carcinoma
Source: Front Oncol. 2022 Jun 8;12:893268. doi: 10.3389/fonc.2022.893268 (PMC9213691; doi:10.3389/fonc.2022.893268)

Supplementary table 1. Clinicopathological characteristics of patients in training cohort and validation cohort.

Variables	Level	Discovery cohort	Validation cohort	P value
Case		617	414	
Age, y	≤50	223 (36.1)	128 (30.9)	0.095
	>50	394 (63.9)	286 (69.1)	
Gender	Male	503 (81.5)	359 (86.7)	0.034
	Female	114 (18.5)	55 (13.3)	
HBsAg	Negative	106 (17.2)	60 (14.5)	0.287
	Positive	511 (82.8)	354 (85.5)	
Cirrhosis	No	263 (42.6)	189 (45.7)	0.370
	Yes	354 (57.4)	225 (54.3)	
Child-Pugh stage	A	616 (99.8)	410 (99.0)	0.172
	B	1 (0.2)	4 (1.0)	
AFP, ng/mL	≤20	242 (39.2)	193 (46.6)	0.022
	>20	375 (60.8)	221 (53.4)	
Tumor size, cm	≤5	393 (63.7)	263 (63.5)	1.000
	>5	224 (36.3)	151 (36.5)	
Tumor number	Solitary	450 (72.9)	317 (76.6)	0.215
	Multiple	167 (27.1)	97 (23.4)	
Edmondson's grade	I/II	361 (58.5)	245 (59.2)	0.881
	III/IV	256 (41.5)	169 (40.8)	
Vascular invasion	No	394 (63.9)	261 (63.0)	0.841
	Yes	223 (36.1)	153 (37.0)	
BCLC stage	A	333 (54.0)	233 (56.3)	0.505
	B/C	284 (46.0)	181 (43.7)	
CNLC stage	I/II	567 (91.9)	392 (94.7)	0.110
	III/IV	50 (8.1)	22 (5.3)	
AJCC stage	I/II	512 (83.0)	347 (83.8)	0.789
	III/IV	105 (17.0)	67 (16.2)	
AST, U/L	≤40	421 (68.2)	289 (69.8)	0.641
	>40	196 (31.8)	125 (30.2)	
ALB, g/L	<35	31 (5.0)	20 (4.8)	1.000
	⩾35	586 (95.0)	394 (95.2)	
GGT, U/L	≤60	361 (58.5)	228 (55.1)	0.304
	>60	256 (41.5)	186 (44.9)	
Lymphocyte, $\times 10^9$/L	median [IQR]	1.70 [1.25, 2.10]	1.70 [1.30, 2.11]	0.277
PLT, $\times 10^9$/L	median [IQR]	151.00 [113.00, 197.00]	159.00 [122.25, 200.75]	0.115
Neutrophil, $\times 10^9$/L	median [IQR]	3.00 [2.30, 3.90]	3.10 [2.32, 4.00]	0.159
CRP, mg/L	median [IQR]	1.40 [0.50, 4.37]	1.70 [0.59, 4.88]	0.175

Supplementary table 2. The formula of existed inflammation-immune models.

Inflammation-immune Models	Score
Glasgow Prognostic Score (GPS)	
CRP (≤10 mg/L) and albumin (≥35 g/L)	0
CRP (≤10 mg/L) and albumin (<35 g/L)	1
CRP (>10 mg/L) and albumin (≥35 g/L)	1
CRP (>10 mg/L) and albumin (<35 g/L)	2
Modified Glasgow Prognostic Score (mGPS)	
CRP (≤10 mg/L) and albumin (≥35 g/L)	0
CRP (≤10 mg/L) and albumin (<35 g/L)	0
CRP (>10 mg/L)	1
CRP (>10 mg/L) and albumin (<35 g/L)	2
Prognostic Nutritional Index (PNI)	
Albumin (g/L) + 5 × lymphocyte count (×10^9/L) ≥45	0
Albumin (g/L) + 5 × lymphocyte count (×10^9/L) <45	1
Neutrophil to lymphocyte ratio (NLR)	
Neutrophil count (×10^9/L): lymphocyte count (×10^9/L) ≤2.7	0
Neutrophil count (×10^9/L): lymphocyte count (×10^9/L) >2.7	1
Platelet to lymphocyte ratio (PLR)	
Platelet count (×10^9/L): lymphocyte count (×10^9/L) ≤133.1	0
Platelet count (×10^9/L): lymphocyte count (×10^9/L) >133.1	1
Systemic immune-inflammation index (SII)	
Platelet count (×10^9/L) × neutrophil count (×10^9/L)/lymphocyte count (×10^9/L) ≤ 523.8	0
Platelet count (×10^9/L) × neutrophil count (×10^9/L)/lymphocyte count (×10^9/L) > 523.8	1
Aspartate aminotransferase to platelet ratio index (APRI)	
AST (U/L): platelet count (×10^9/L) <0.62	0
AST (U/L): platelet count (×10^9/L) ⩾0.62	1
Aspartate aminotransferase to lymphocyte ratio index (ALRI)	
AST (U/L): lymphocyte count (×10^9/L) ⩽25.2	0
AST (U/L): lymphocyte count (×10^9/L) >25.2	1

CRP: C-reactive protein; AST: Aspartate aminotransferase.

Supplementary table 3. The formula of nine candidate models.

Models	Formula
ACLR	AST (U/L) × CRP (mg/L)/lymphocyte count (×10^9/L)
NALR	Neutrophil count (×10^9/L) × AST (U/L)/lymphocyte count (×10^9/L)
NCLR	Neutrophil count (×10^9/L) × CRP (mg/L)/lymphocyte count (×10^9/L)
ACBR	AST (U/L) × CRP (mg/L)/albumin (g/L)
NABR	Neutrophil count (×10^9/L) × AST (U/L)/albumin (g/L)
NCBR	Neutrophil count (×10^9/L) × CRP (mg/L)/albumin (g/L)
ALBR	AST (U/L)/ (lymphocyte count (×10^9/L) × albumin (g/L))
NLBR	Neutrophil count (×10^9/L)/ (lymphocyte count (×10^9/L) × albumin (g/L))
CLBR	CRP (mg/L)/ (lymphocyte count (×10^9/L) × albumin (g/L))

AST: Aspartate aminotransferase; CRP: C-reactive protein

Supplementary table 4. Univariate and multivariate analyses in validation cohort.

Variables	OS				TTR			
	Univariate analysis		Multivariate analysis		Univariate analysis		Multivariate analysis	
	HR (95%CI)	***P* value**	HR (95%CI)	***P* value**	HR (95%CI)	***P* value**	HR (95%CI)	***P* value**
Age, y (50: >50)	0.78 (0.53-1.14)	0.202			0.74 (0.57-0.96)	0.024	0.87 (0.66-1.15)	0.333
Gender (male: female)	0.66 (0.35-1.23)	0.187			0.73 (0.49-1.09)	0.121		
HBsAg (negative: positive)	0.60 (0.38-0.94)	0.026	0.62 (0.38-1.01)	0.056	1.25 (0.85-1.83)	0.253		
Cirrhosis (no: yes)	1.16 (0.80-1.67)	0.447			1.15 (0.89-1.48)	0.290		
Child-Pugh stage (A: B)	6.12 (1.94-19.31)	0.002	6.05 (1.68-21.75)	0.006	3.87 (1.23-12.14)	0.021	2.71 (0.82-9.00)	0.104
AFP, ng/mL (≤20: >20)	2.88 (1.90-4.37)	<0.001	2.23 (1.42-3.51)	<0.001	1.73 (1.34-2.23)	<0.001	1.50 (1.14-1.98)	0.004
Tumor size, cm (≤5: >5)	4.53 (3.07-6.68)	<0.001	1.73 (1.07-2.78)	0.024	2.47 (1.92-3.18)	<0.001	1.46 (1.08-1.97)	0.013
Tumor number (solitary: multiple)	2.27 (1.55-3.32)	<0.001	1.59 (1.05-2.40)	0.027	2.29 (1.74-3.01)	<0.001	1.83 (1.37-2.46)	<0.001
Edmondson grade (I/II: III/IV)	2.73 (1.88-3.98)	<0.001	1.59 (1.06-2.39)	0.024	1.82 (1.41-2.34)	<0.001	1.26 (0.96-1.66)	0.091
Vascular invasion (no: yes)	2.88 (1.98-4.18)	<0.001	1.36 (0.89-2.06)	0.154	2.32 (1.80-2.99)	<0.001	1.49 (1.12-1.98)	0.006
GPS	2.95 (2.22-3.92)	<0.001	1.06 (0.74-1.53)	0.750	1.99 (1.55-2.54)	<0.001	1.08 (0.79-1.47)	0.625
NLR (≤2.7: >2.7)	2.96 (2.03-4.33)	<0.001	1.86 (1.06-3.25)	0.030	1.81 (1.36-2.43)	<0.001	1.46 (0.98-2.15)	0.061
PLR (≤133.1: >133.1)	2.19 (1.47-3.25)	<0.001	0.60 (0.37-0.98)	0.040	1.27 (0.93-1.73)	0.131		
SII (≤523.8: >523.8)	3.52 (2.40-5.17)	<0.001	1.35 (0.73-2.48)	0.337	1.90 (1.40-2.58)	<0.001	1.05 (0.69-1.59)	0.822
ACLR (≤80: >80)	6.91 (4.67-10.24)	<0.001	4.16 (2.59-6.69)	<0.001	3.25 (2.51-4.21)	<0.001	2.19 (1.59-3.03)	<0.001

Supplementary Figure 1. Schematic chart for the combination of inflammatory factors in this study. Five inflammatory factors were selected and combined to find the model with the highest accuracy to predict prognosis in HCC patients.

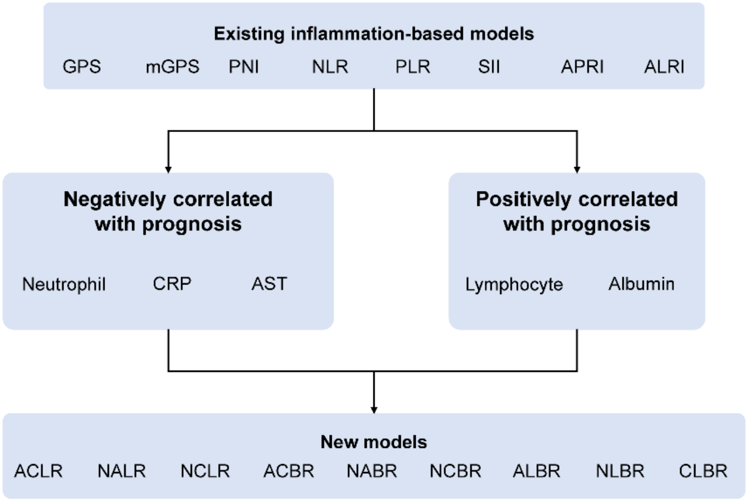

Supplementary Figure 2. Correlation of ACLR with clinical features in HCC patients from the training cohort. Scattergrams of ACLR according to (A) tumor size, (B) Edmondson grade, (C) vascular invasion and (D) AFP level in HCC patients from training cohort.

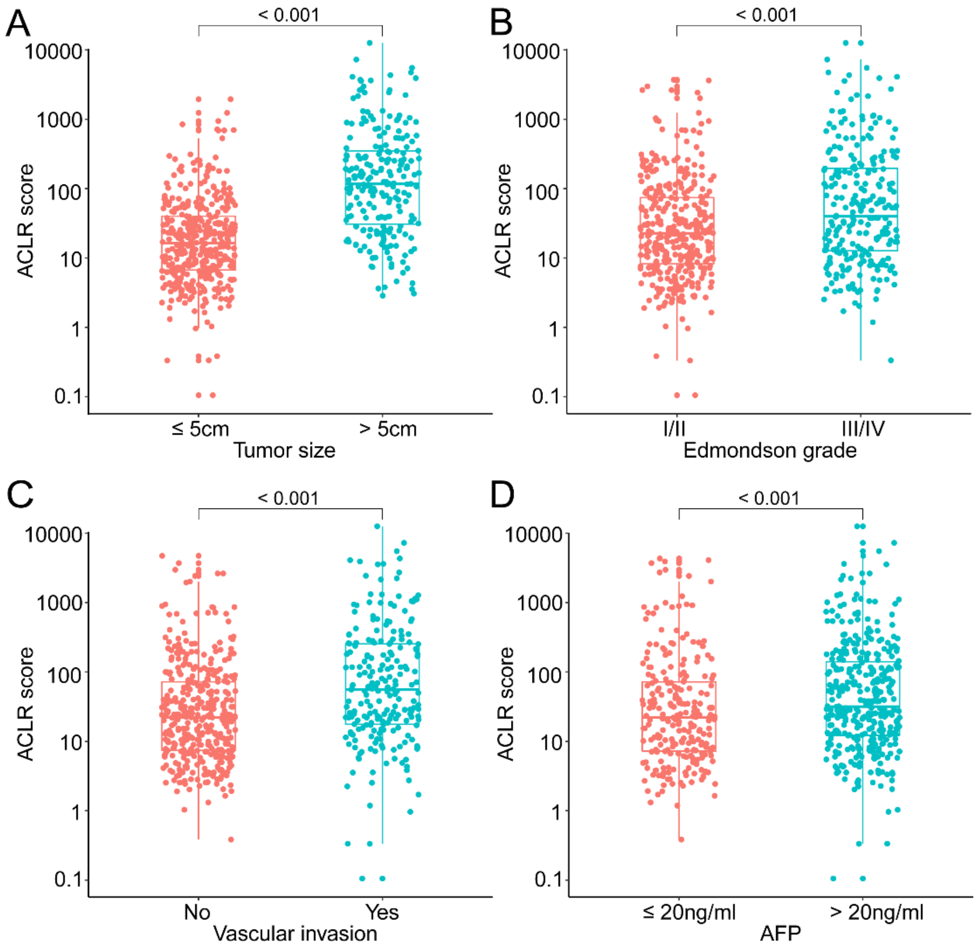

Supplementary Figure 3. Correlation of ACLR with clinical features in HCC patients from the validation cohort. Scattergrams of ACLR according to (A) tumor size, (B) Edmondson grade, (C) vascular invasion and (D) AFP levelin HCC patients from validation cohort.

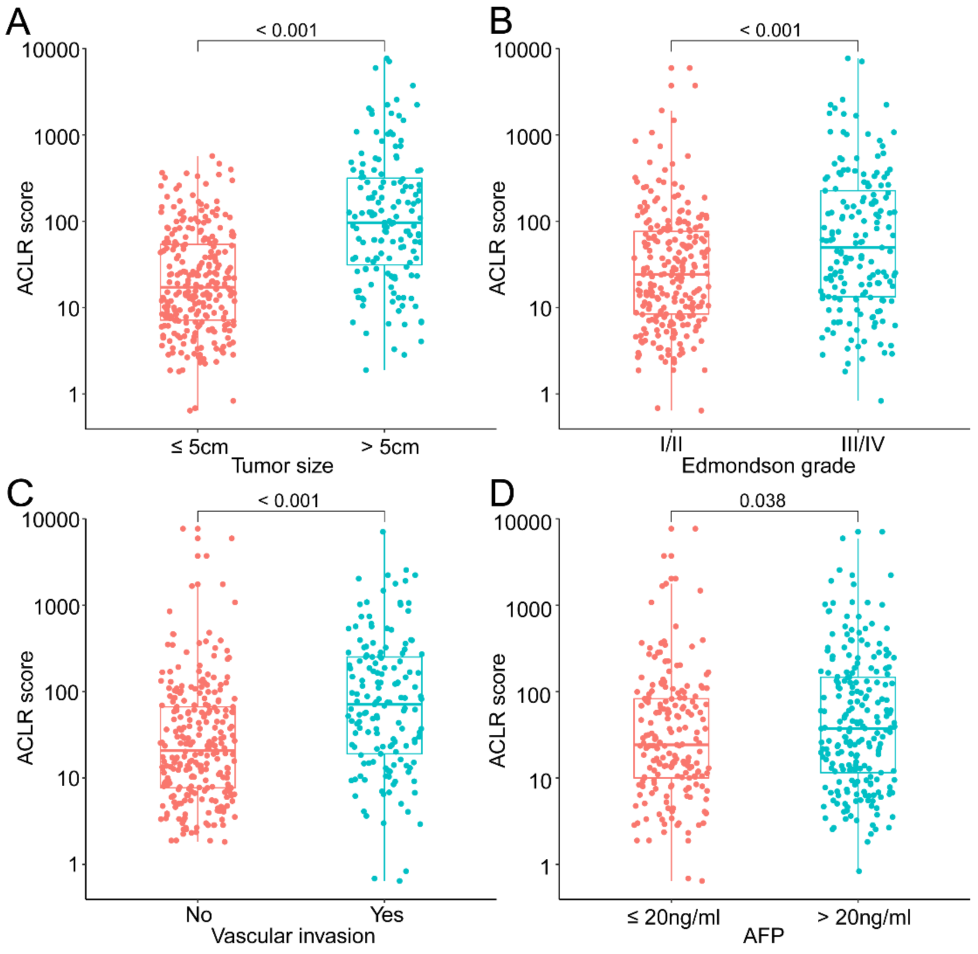

Supplementary Figure 4. Impact of surgical margin for prognosis of HCC patients from the whole cohort. (A-C) Kaplan-Meier analysis of 2-year recurrence rate (A), TTR (B) and OS (C) for HCC patients, stratified by surgical margin.

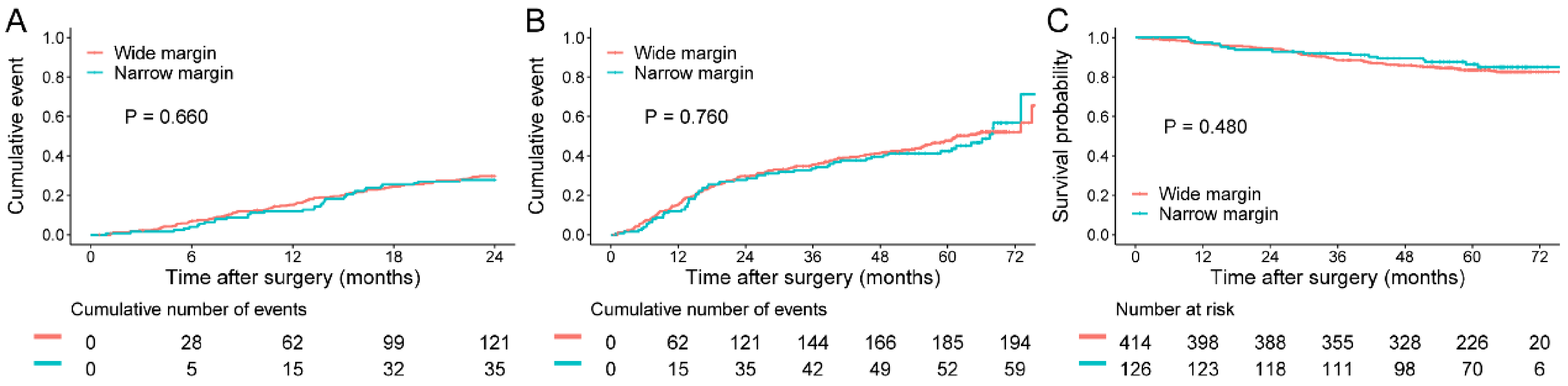

Supplementary Figure 5. Correlation between resection margin and recurrence, stratified by ACLR. (A) Recurrence around resection margin in patients with high and low ACLR; (B) at resection margin ≤ 1 cm, high ACLR correlated with higher risk of margin recurrence; (C) at resection margin > 1 cm, no difference in the margin recurrence risk was observed between high and low ACLR patients.

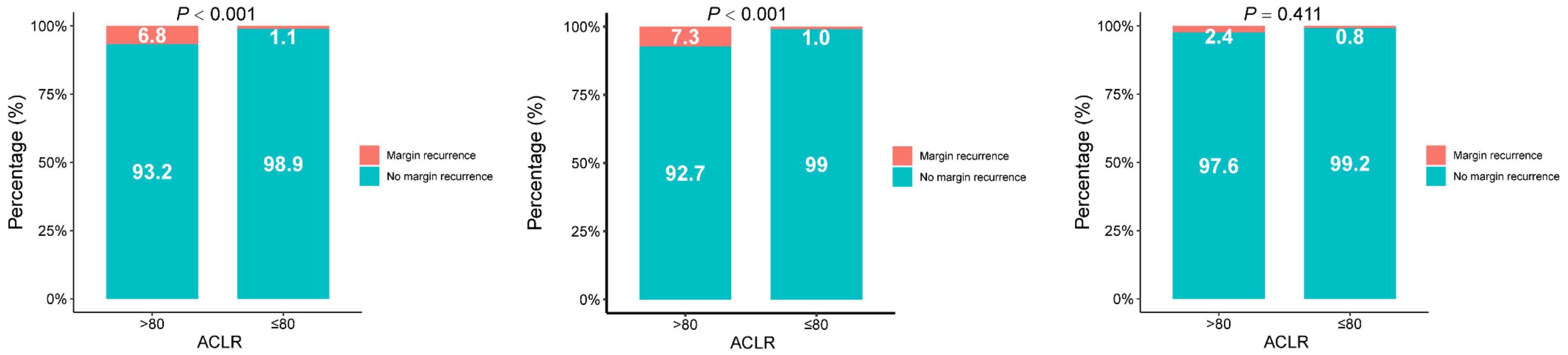

Supplement: Supplementary file 1 [file DataSheet_1.pdf]
